# Large-scale genetic correlation scanning and causal association between deep vein thrombosis and human blood metabolites

**DOI:** 10.1038/s41598-022-12021-x

**Published:** 2022-05-12

**Authors:** Pan Luo, Jiawen Xu, Shiqiang Cheng, Ke Xu, Wensen Jing, Feng Zhang, Peng Xu

**Affiliations:** 1 Department of Joint Surgery, HongHui Hospital, Xi’an Jiaotong University, Xi’an, 710054 Shanxi China; 2 Orthopedic Research Institute, Department of Orthopedics, West China Hospital, Sichuan University, 37# Guoxue Road, Chengdu, 610041 People’s Republic of China; 3 Key Laboratory of Trace Elements and Endemic Diseases, National Health and Family Planning Commission, School of Public Health, Health Science Center, Xi’an Jiao Tong University, No.76 Yan Ta West Road, Xi’an, 710061 People’s Republic of China

**Keywords:** Genetics, Diseases

## Abstract

Deep vein thrombosis (DVT) refers to the abnormal coagulation of blood in a deep vein. Recently, some studies have found that metabolites are related to the occurrence of DVT and may serve as new markers for the diagnosis of DVT. In this study, we used the GWAS summary dataset of blood metabolites and DVT to perform a large-scale genetic correlation scan of DVT and blood metabolites to explore the correlation between blood metabolites and DVT. We used GWAS summary data of DVT from the UK Biobank (UK Biobank fields: 20002) and GWAS summary data of blood metabolites from a previously published study (including 529 metabolites in plasma or serum from 7824 adults from two European population studies) for genetic correlation analysis. Then, we conducted a causal study between the screened blood metabolites and DVT by Mendelian randomization (MR) analysis. In the first stage, genetic correlation analysis identified 9 blood metabolites that demonstrated a suggestive association with DVT. These metabolites included Valine (correlation coefficient = 0.2440, P value = 0.0430), Carnitine (correlation coefficient = 0.1574, P value = 0.0146), Hydroxytryptophan (correlation coefficient = 0.2376, P value = 0.0360), and 1-stearoylglycerophosphoethanolamine (correlation coefficient = − 0.3850, P value = 0.0258). Then, based on the IVW MR model, we analysed the causal relationship between the screened blood metabolites and DVT and found that there was a suggestive causal relationship between Hydroxytryptophan (exposure) and DVT (outcome) (β = − 0.0378, se = 0.0163, P = 0.0204). Our study identified a set of candidate blood metabolites that showed a suggestive association with DVT. We hope that our findings will provide new insights into the pathogenesis and diagnosis of DVT in the future.

## Introduction

Deep vein thrombosis (DVT) refers to the abnormal coagulation of blood in a deep vein. It leads to the obstruction of blood reflux and causes lower extremity oedema and even pulmonary embolism^1,2^. In addition, DVT is an important complication of several inherited and acquired diseases, but it can also occur spontaneously^3^. Currently, there are many methods for the diagnosis of DVT, such as d-dimer and ultrasound (US) imaging, that have been adopted to aid in the diagnosis of DVT pathways^4^. Recently, some studies have found that metabolites are related to the occurrence of DVT and may be new markers for the diagnosis of DVT^5^.

The metabolome is defined as the collection of metabolites and small molecules that are involved in cell metabolism. They are produced in cells and can be divided into many categories^6^. Approximately 50% of the total phenotypic variation in metabolite levels is due to SNP, but estimates of heritability vary by metabolite class^7^. Genomic and metabolomic analyses of common SNPs in human metabolism have successfully identified the metabolites affected by genetics^8^. The elucidation of the genetic mechanism of metabolism may provide new therapeutic targets or new biomarkers for disease diagnosis^9^. Among them, metabolism in human blood is controlled by different degrees of genetic effects, complex regulatory effects and nongenetic effects^10^.

The genetic control of metabolite levels and their impact on human health is evident in inborn metabolic errors. In these errors, rare SNPs disrupt individual genes and then lead to extreme and ultimately toxic levels of the related metabolites^8^. Genome-wide association studies with metabolomics (mGWAS) that use population-scale metabolomics and genotypic data can systematically study the less obvious effects of more common and less harmful SNPs on human metabolism. This was demonstrated by Gieger et al. in the first mGWAS^11^.

Genetic correlation is an important population parameter that can describe the genetic relationships between two traits^12–14^. Using summary data from genome-wide association studies (GWAS), LDSC can screen for thousands of traits simultaneously and find genetic correlations between them^13^. Mendelian randomization (MR) refers to studies in observational epidemiology that use SNP to make causal inferences about risk factors for disease and health-related outcomes^15,16^. MR analysis presents a valuable tool, especially when randomized controlled trials to examine causality are not feasible and observational studies provide biased associations because of confounding or reverse causality. These issues are addressed by using genetic variants as instrumental variables for the tested exposure: the alleles of this exposure-associated genetic variant are randomly allocated and not subject to reverse causation^17^. Previous studies have used LDSC and MR to analyse the association between inflammation pathway and suicide and found that IL-6 signalling is associated with suicide^18^. Thus, we hope to use LDSC and MR analysis to further dissect the association between blood metabolites and DVT.

In this study, for the first time, we used a large-scale GWAS summary dataset of blood metabolites and DVT to perform a genetic correlation scan of DVT and blood metabolites to explore the genetic relationship between blood metabolites and DVT. Our study has the potential to provide new insights into the genetic mechanisms, diagnosis and treatment of DVT.

## Methods

### GWAS summary datasets of DVT

The GWAS summary data of DVT used in this study were obtained from the UK Biobank (UK Biobank fields: 20002)^19^. The DVT cases in the UK Biobank were defined based on self-reported diagnosis. The UK Biobank was a large prospective cohort study involving approximately 500,000 people aged 37 to 76 years (99.5% aged 40 to 69 years) from across the UK. This cohort included 9059 DVT patients and 443,205 control cases^20^. The UK Biobank has received ethical approval from the Northwest Multicentre Research Ethics Committee and the informed consent of all participants. All participants provided a range of information on their health status, demographics and lifestyle through questionnaires and interviews^19^. Detailed information on the samples, imputation and genotyping can be found in previously published studies^19^.

### GWAS summary datasets of human blood metabolites

A previously published large-scale GWAS dataset of human blood metabolites was used here^10^. The study sample included 529 metabolites in the plasma or serum from 7824 adults from two European population studies^10^. More than half of the 529 metabolites (N = 333, 63%) can be chemically identified as 8 metabolic groups (amino acids, carbohydrates, cofactors and vitamins, energy, fat, nucleotides, peptides and xenobiotics)^10^. After strict quality control, there were 486 subsets of metabolites available for genetic analysis, including 309 known metabolites and 177 unknown metabolites^10^. Detailed descriptions of the quality control, sample characteristics, research design and statistical analysis can be found in previously published studies^10^.

### Statistical analysis

Referring to the methods recommended by the developers^13,14^ and previous studies^21^, we used LDSC software (v1.0.0; https://github.com/bulik/ldsc) to analyse the genetic correlation between each blood metabolite and DVT. The basic principle of the LDSC method is to estimate the deviation between the χ^2^ test statistics of an SNP and its expected values directly from the GWAS summary data under the null hypothesis of no association^22^. The study used the European LD score, which was calculated by the developers from 1000 genomes^23^. After correcting for multiple testing, the significance threshold of this study should be P < 9.45 × 10^−5^ (0.05/529 = 9.45 × 10^−5^). Since few metabolites reached the significance level after multiple test corrections, P < 0.05 was adopted as the suggested significance level.

MR analysis can be used to evaluate the causal relationship between blood metabolites and DVT. The MR technique uses SNPs related to modifiable traits/exposures as tools to detect causal associations among the outcomes^15^. In an MR test, three key assumptions must be met: (1) the SNP is directly associated with the exposure; (2) the SNP is not related to factors known to obscure the connection between the exposure and the outcome; and (3) the SNP has no effect on outcome. The inverse-variance weighted (IVW) method uses a meta-analysis approach to combine the Wald ratio estimates of the causal effect obtained from different SNPs and to provide a consistent estimate of the causal effect of the exposure on the outcome when each of the SNPs satisfies the assumptions of an instrumental variable^24^. Egger regression is a tool to detect small study bias in meta-analysis and can be adapted to test for bias from pleiotropy. The slope coefficient from Egger regression provides an estimate of the causal effect^25^. The weighted median estimate provides a consistent estimate of causal effects, even if up to 50 percent of the analytical information comes from SNP of ineffective IVs^26^. In addition, MR-Egger and weighted median can be used as sensitivity analysis. Particularly, MR-Egger and weighted median could provide a more valid MR estimates if their assumptions are met and if multiple SNPs are pleiotropic. To test whether there was a weak instrumental variable bias, namely genetic variants selected as instrumental variables had a weak association with exposure, we calculated the F statistic (F = R2(n − k − 1)/k(1 − R2); R2, variance of exposure explained by selected instrumental variables, and we got the value of R2 in MR Steiger directionality test; n, sample size; and k, number of instrumental variables). If the F statistic is much greater than 10 for the instrument-exposure association, the possibility of weak instrumental variable bias is small.

In the absence of pleiotropy, the IVW estimator is the gold standard method. The main analysis was IVW, and the sensitivity analysis was the Leave-one-out test. When the effect SNP of the metabolite is more than 3, we test the MR results by Leave-one-out test. We used the Wald estimator when there is only one instrument available, the IVW method when at least two instruments were selected, and the IVW, the MR-Egger, and the weighted median methods with more than 3 instruments.

We used the MR basic platform (http://app.mrbase.org/) to analyse the causal relationship between the screened blood metabolites and DVT. After correcting for multiple testing, the significance threshold of this study should be P < 0.005 (0.05/9 = 0.005). Since few metabolites reached the significance level after multiple test corrections, P < 0.05 was adopted as the suggested significance level.

All methods were performed in accordance with the relevant guidelines and regulations (for example—Declarations of Helsinki).

## Results

**Figure 1:**
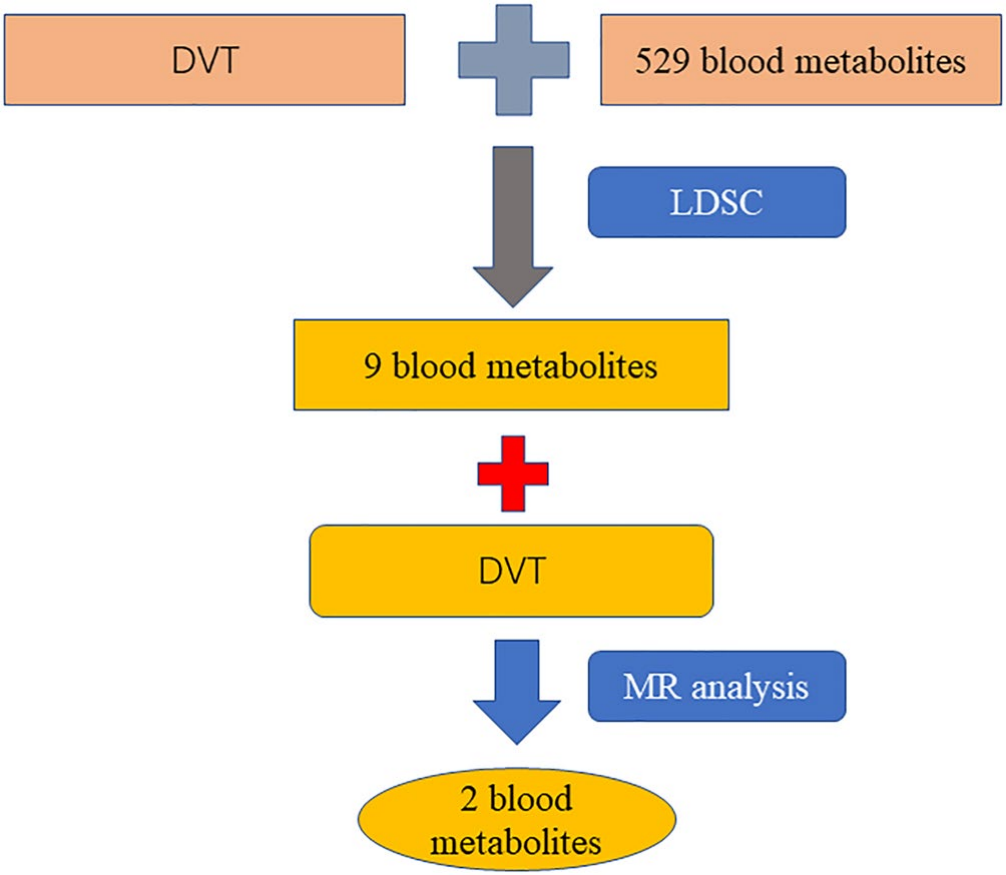
Detailed flowchart of the result. We analyzed the genetic correlation between DVT and 529 blood metabolites by LDSC, and found that 9 blood metabolites may had genetic correlation with DVT. Then the causal relationship between these 9 blood metabolites and DVT was analyzed by MR, and it was found that 2 blood metabolites had causal correlation with DVT.

In the first stage, genetic correlation analysis identified 9 suggestive blood metabolites that were significantly associated with DVT (Fig. 1, Table 1), including Valine, Carnitine, Hydroxytryptophan, 1-stearoylglycerophosphoethanolamine, X-11317, X-11550, X-12465, X-12644, and X-13741. The LDSC results of those that do not reach the level of significance are shown in the Supplementary Table S1 and Supplementary Table S2.

**Table 1:** Genetic correlation between human blood metabolites and deep vein thrombosis.

	Blood metabolites	Gene	Genetic correlation	P value
Deep vein thrombosis	Valine	PPM1K	0.2440	0.0430
	Carnitine	PEX5L	0.1574	0.0146
	X-11317		− 0.2181	0.0377
	X-11550	SLC5A11	− 0.2855	0.0435
	Hydroxytryptophan	IDO1	0.2376	0.0360
	X-12465		0.3643	0.0353
	X-12644		− 0.4936	0.0057
	1-stearoylglycerophosphoethanolamine		− 0.3850	0.0258
	X-13741		0.6743	0.0285

LD score regression software (https://github.com/bulik/ldsc) was used here to evaluate the genetic correlation between deep vein thrombosis and each of the human blood metabolites. So far, no genes related to the other five blood metabolites (X-11317, X-12465, X-12644, 1-stearoylglycerophosphoethanolamine, X-13741) have been found.

**Figure 2:**
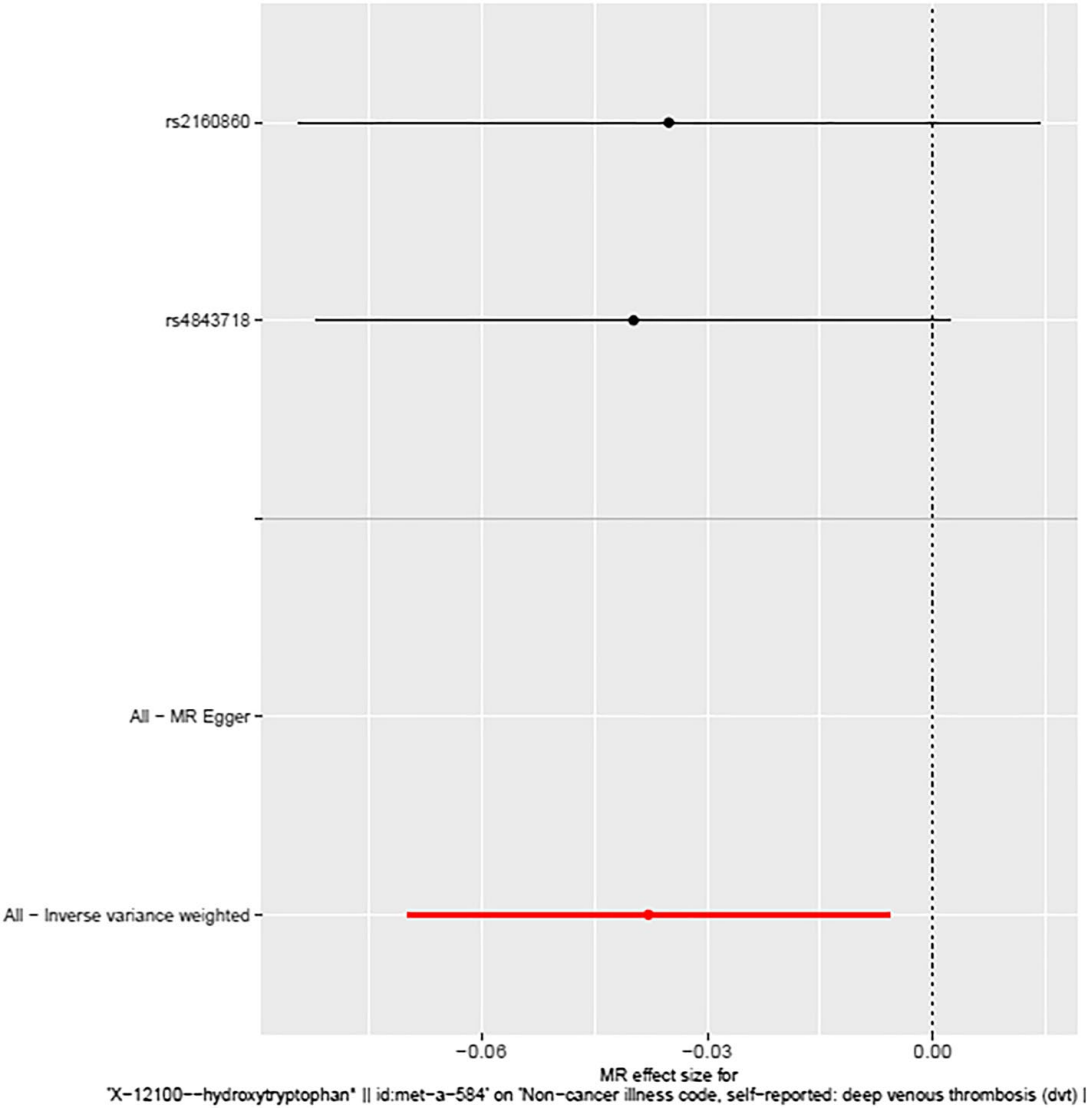
Forest Map of causality between Hydroxytryptophan-related SNP and DVT. The causal effect of exposure on outcome is estimated using each SNP singly using the Wald ratio, and represented in a forest plot. The MR estimate using all SNPs using the MR Egger and IVW methods are also shown.

**Figure 3:**
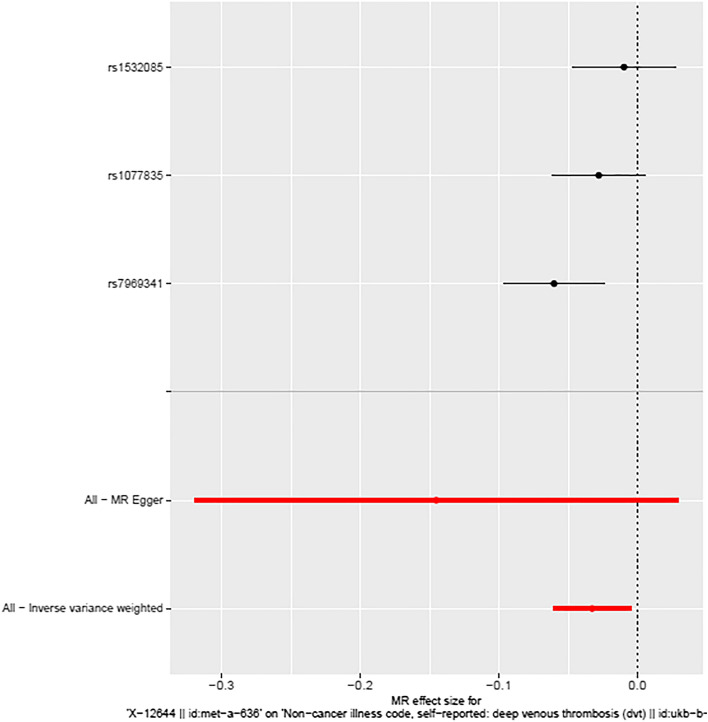
Forest Map of causality between X-12644-related SNP and DVT. The causal effect of exposure on outcome is estimated using each SNP singly using the Wald ratio, and represented in a forest plot. The MR estimate using all SNPs using the MR Egger and IVW methods are also shown.

**Figure 4:**
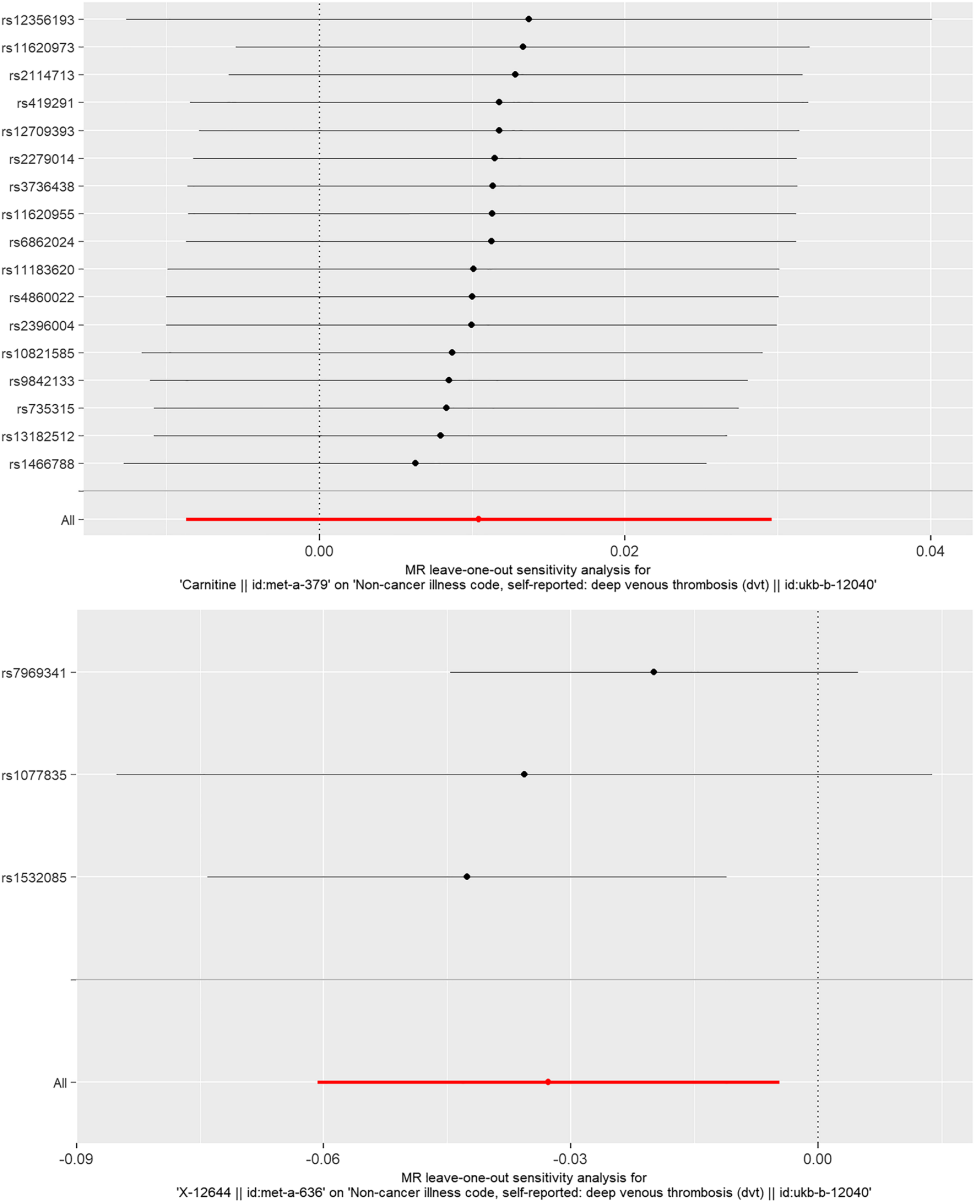
Sensitivity analysis of the MR results (Carnitine, X-12644). As the other 7 blood metabolites were included in fewer instruments, the Leave-one-out test could not be used to test them.

We conducted instrumental variable screening strictly in accordance with the three core assumptions of Mendelian randomization analysis. First, SNP must be strongly correlated with exposure factor, so we screened SNPs with P < 5 × 10^−8^, R2 = 0.001, KB = 10,000. We used the PhenoScanner platform to exclude SNPs associated with DVT and risk factors associated with DVT to show that there is no horizontal pleiotropy in our analysis from a biological point of view. Based on the IVW MR model, we analysed the causal relationship between the screened blood metabolites and DVT and found that there was a suggestive inverse causal relationship between Hydroxytryptophan (exposure) and DVT (outcome) (Table 2, Fig. 2). In addition, we also found that there was a suggestive inverse causal relationship between X-12644 (exposure) and DVT (outcome) (Table 2, Fig. 3). The Egger intercept did not deviate significantly from zero (intercept = 0.0021, SE = 0.0017, P = 0.423). Thus, there was no evidence for unbalanced pleiotropy, which would suggest that the IVW estimates were unbiased. However, the results of the MR–Egger and weighted median methods did not support this conclusion (MR–Egger: β = − 0.1452, se = 0.0889, P = 0.3498; weighted median: β = − 0.0265, se = 0.0146, P = 0.0694). In addition, the difference between Q and Q′ (Q − Q′ = 0.2327) is not sufficiently extreme under a χ_1_^2^ distribution, which means that the MR–Egger model does not fit our data better than the IVW model. Because some metabolites included fewer instruments, we evaluated the instruments included in Table 3. According to the above results, we found that the F values of the instrumental variables included in X-12644 and Hydroxytryptophan were all more than 10, which effectively reduce the likelihood of weak instrument bias. In addition, sensitivity analysis was performed on the results (leave-one-out test). As the other 7 blood metabolites were included in fewer instruments, the leave-one-out test could not be used to test them. Only Carnitine and X-12644 were subjected to the leave-one-out method for sensitivity analysis. The results are shown in Fig. 4. According to the result of the leave-one-out test, we found that the MR analysis of Carnitine was stable, while the MR analysis of X-12644 was not. (Fig. 4).

**Table 2:** The results of causal analysis of human blood metabolites (exposure) and deep vein thrombosis (outcome).

Exposure group	Outcome group	Instruments	Analytical method	Beta	SE	P
Valine	DVT	rs1440581	Wald ratio	− 0.0477	0.0359	0.1847
Carnitine	DVT	rs735315 rs419291 rs4860022 rs11183620 rs1466788 rs2279014 rs10821585 rs11620955 rs12709393 rs13182512 rs12356193 rs6862024 rs2114713 rs9842133 rs2396004 rs11620973 rs3736438	MR Egger	0.0115	0.0189	0.5499
			Weighted median	0.0079	0.0133	0.5505
			Inverse variance weighted	0.0104	0.0097	0.2859
X-11317	DVT	rs7797368 rs7499892	Inverse variance weighted	− 0.0096	0.0153	0.5300
X-11550	DVT	rs247616	Wald ratio	− 0.0325	0.0324	0.3155
Hydroxytryptophan	DVT	rs4843718 rs2160860	Inverse variance weighted	− 0.0378	0.0163	**0.0204**
X-12644	DVT	rs1532085 rs1077835 rs7969341	MR Egger	− 0.1452	0.0889	0.3498
			Weighted median	− 0.0265	0.0146	0.0694
			Inverse variance weighted	− 0.0327	0.0142	**0.0219**
1-stearoylglycerophosphoethanolamine	DVT	rs588136	Wald ratio	− 0.0206	0.0121	0.0901
X-13741	DVT	rs12189736	Wald ratio	− 0.0045	0.0072	0.5341

Since no SNP in X-12465 can perform causal analysis, it is not listed.

DVT: deep vein thrombosis.

Significant values are in bold.

**Table 3:** The description of the instruments.

	Instruments	F	R2
X-11317	rs7797368	3.71	0.00095
	rs7499892	1.09	0.00028
Valine	rs1440581	15	0.00194
Carnitine	rs735315	0.61	0.00135
	rs419291	0.26	0.00058
	rs4860022	0.23	0.00052
	rs11183620	1.14	0.00249
	rs1466788	0.29	0.00064
	rs2279014	0.43	0.00095
	rs10821585	1.2	0.00263
	rs11620955	1.35	0.00296
	rs12709393	0.92	0.00202
	rs13182512	0.34	0.00076
	rs12356193	0.32	0.00070
	rs6862024	0.20	0.00045
	rs2114713	0.17	0.00039
	rs9842133	0.29	0.00064
	rs2396004	0.19	0.00043
	rs11620973	0.97	0.00212
	rs3736438	0.82	0.00180
X-11550	rs247616	3.83	0.00147
Hydroxytryptophan	rs4843718	8.01	0.00205
	rs2160860	10.6	0.00272
X-12644	rs1532085	1.95	0.00075
	rs1077835	6.33	0.00243
	rs7969341	12.4	0.00478
1-stearoylglycerophosphoethanolamine	rs588136	19.4	0.00249
X-13741	rs12189736	7.11	0.00091

Then, we performed a reverse MR analysis. However, when DVT was used as the exposure variable, we found no causal relationship between DVT and 9 blood metabolites, as shown in Table 4.

**Table 4:** The results of causal analysis of deep vein thrombosis (exposure) and human blood metabolites (outcome).

Exposure group	Outcome group	SNP	Analytical method	P value
DVT	Valine	rs687289 rs2066865 rs867186 rs10838599 rs17490626 rs4253399	IVW	0.421
DVT	Carnitine	rs4253399 rs2066865 rs10838599 rs17490626 rs867186 rs687289	IVW	0.765
DVT	X-11317	rs17490626 rs10838599 rs867186 rs687289 rs2066865 rs4253399	IVW	0.437
DVT	X-11550	rs4253399 rs687289 rs867186 rs17490626 rs10838599 rs2066865	IVW	0.658
DVT	Hydroxytryptophan	rs10838599 rs687289 rs4253399 rs2066865 rs867186 rs17490626	IVW	0.658
DVT	X-12644	rs867186 rs17490626 rs2066865 rs10838599 rs687289 rs4253399	IVW	0.267
DVT	1-stearoylglycerophosphoethanolamine	rs4253399 rs2066865 rs867186 rs17490626 rs10838599 rs687289	IVW	0.905
DVT	X-13741	rs10838599 rs687289 rs17490626 rs4253399 rs867186 rs2066865	IVW	0.171
DVT	X-12465	rs2066865 rs10838599 rs687289 rs17490626 rs4253399 rs867186	IVW	0.198

DVT, deep vein thrombosis.

## Discussion

DVT is a disease that is affected by many factors and is caused by the interaction of a series of acquired and hereditary risk factors^27^. Major hereditary thrombotic diseases include a lack of natural anticoagulants, antithrombin and proteins C and S in plasma^28^. The clinical diagnosis of DVT mainly depends on clinical symptoms, d-dimer levels and ultrasonic examination^29,30^. Although the plasma level of d-dimer is highly sensitive, its deletion may help to exclude DVT^31^. However, d-dimer levels are easily altered by cancer, surgery and other factors, so its specificity and positive predictive value are very low^32^. Therefore, more appropriate biomarkers are needed to conduct appropriate risk assessment for the occurrence of DVT to improve the sensitivity of DVT diagnosis, timely treatment or avoid invasive surgery. As an important part of systems biology^33^, metabonomics has been widely used in the study of pathogenesis-related diagnostic and prognostic biomarkers by detecting endogenous small molecule compounds in biological samples^34,35^. Recent studies have found that glycolysis, purines and redox-related metabolites may contribute to the discovery of fresh venous thrombosis, and changes in metabolites may affect the formation of venous thrombosis^5^. Therefore, we hope to provide new ideas for the diagnosis of DVT and provide insights into the genetic mechanisms of DVT by studying the relationship between blood metabolites and DVT.

To study the relationship between blood metabolites and DVT, we carried out genetic correlation analysis based on the GWAS summary data of blood metabolites and DVT. We found that 9 blood metabolites were genetically correlated with DVT, including 4 known metabolites and 5 unknown metabolites. Then, we analysed the identified blood metabolites and DVT by MR and found that there was an inverse causal relationship between the two blood metabolites and DVT. To our knowledge, this is the first time that a large-scale genetic association between blood metabolites and DVT has been assessed, and our findings may greatly expand the biological knowledge associated with DVT.

Valine, also known as 2-amino-3-methylbutyric acid, is a branched-chain amino acid. It is one of the eight essential amino acids and sugar-producing amino acids of the human body^36^. It can promote normal growth, repair tissue, regulate blood sugar and provide the necessary energy^37^. A mutation in the gene for Factor XIII that leads to a Valine-leucine exchange has been reported to be protective against DVT^38^. Factor XIII (FXIII) is a transglutaminase found in plasma and platelets^39^. During thrombus formation, activated FXIII cross-links fibrin, which promotes thrombus stability^39^. Fujimura et al. found that serum samples from patients with DVT showed high levels of Valine^40^. FXIII cross-links fibrin when thrombin is activated. The activation of thrombin releases activated peptides. A common polymorphism (Valine to leucine variant at residue 34, V34L) that is located in the activating peptide has been found to be associated with the prevention of thrombosis^41^. However, the specific mechanism by which Valine participates in the prevention of thrombi is still unclear and requires further experimental research.

Carnitine is considered a conditionally essential nutrient because of its importance in human physiology^42^. The anticoagulant effect of Carnitine is related to the regulation of prostaglandin formation by its derivatives, which can stimulate prostacyclin production^43^. The cytoprotective and vasodilator effects of prostacyclin are well known. It has been shown that l-Carnitine supplementation reduces serum CRP and plasma fibrinogen levels in haemodialysis patients^44^. Supplementation with l-Carnitine reduces inflammatory substances, such as CRP, IL-6, and TNF-α, and increases oxidative stress levels^45^. Deguchi et al. confirmed that there is a low level of acyl Carnitine in the plasma of patients with venous thromboembolism. They also showed that acyl Carnitine can act as an anticoagulant because of its ability to bind and inhibit Xa factor^46^. Our study found a genetic correlation between Carnitine and DVT, which agrees with these previous studies. Moreover, the genetic association between DVT and Carnitine found in our study provides a new direction for further research on how Carnitine affects the pathogenesis of DVT.

For the results of the LDSC, DVT was found to be genetically correlated with Valine and Carnitine. We hold the opinion that these results should be interpreted as Valine and Carnitine being genetically correlated with DVT. However, these two metabolisms may not lead to deep vein thrombosis, and specific directions should be explained by referring to relevant literature. Through a literature search, we found no evidence that Valine and Carnitine may be involved in increasing the incidence of DVT. Instead, we found that these two metabolites are involved in anticoagulation, which needs to be stated.

5-Hydroxytryptophan (5-HTP) is a kind of amino acid. It can be used as a precursor of serotonin (serotonin, 5-HT) in the human body (and then as a precursor of melatonin)^47^. According to clinical studies, taking 5-HTP can significantly improve the mood of patients with depression^48,49^. Studies have found that serotonergic antidepressants have a weak anticoagulant effect^50^. In addition, selective serotonin reuptake inhibitors (SSRIs) inhibit the formation of tight clots of platelets in vitro, which indicates that SSRIs have a direct antithrombotic or fibrinolytic effect^51^. Serotonin stored in platelets accounts for more than 99% of the total serotonin concentration in the human body. After blood vessel injury and platelet activation, serotonin is released into the bloodstream and binds to specific receptors. It thereby promotes vasoconstriction and platelet aggregation and facilitates haemostasis^52^. Serotonin reuptake into platelets involves a serotonin transporter that is blocked by the SSRI, thereby inhibiting serotonin reuptake into platelets^53^. This inhibition in turn reduces the likelihood of platelet agglutination. This then reduces the formation of platelet thrombosis, which in turn increases the risk of bleeding^53,54^. We found that DVT and 5-HTP were genetically correlated, and 5-HTP and DVT demonstrated reverse causality. However, no studies have determined how 5-HTP is involved in the pathogenesis of DVT. In the analysis results of the LDSC, we found that hydroxyl tryptophan and DVT have a strong genetic correlation, but in patients with DVT, the hydroxyl tryptophan levels may be higher than normal. This is not just because of the disease itself but also because of genetic factors. Thus, it cannot explain why Hydroxytryptophan causes DVT. In contrast, MR analysis found that Hydroxytryptophan was negatively correlated with DVT. Our study provides a new idea for the involvement of 5-HTP in the pathogenesis and diagnosis of DVT.

According to our study, another 6 blood metabolites were found to be genetically correlated with DVT, and among them, X-12644 had a reverse causal relationship with DVT. However, we have not found any studies on the correlation between these blood metabolites and DVT or blood coagulation.

To the best of our knowledge, this is the first large-scale genetic correlation analysis of the blood metabolic groups and DVT. Because we used GWAS gene data, the results are not easily affected by environmental confounding factors. Furthermore, we not only studied the genetic correlation between DVT and blood metabolites but also determined the causal relationship. Of course, some limitations of this research should be noted. First, the significance threshold should be P < 9.45 × 10^−5^ after multiple test correction. Unfortunately, according to our results, there was no significant genetic correlation at this threshold. Since all blood metabolites identified in this study are suggested to be associated with DVT, the results should be interpreted carefully. Furthermore, it should be noted that the purpose of this study was to evaluate the genetic correlation between blood metabolites and DVT and to scan for new candidate blood metabolites associated with DVT. In this study, further basic research is needed to confirm our findings and to clarify the potential biological mechanism of the observed link between blood metabolites and DVT. Finally, the GWAS summary data of this study are all from European origin. Therefore, we should be careful to apply our research results to other ethnic groups.

## Conclusion

In short, by using the LDSC method, we conducted a large-scale analysis to investigate the genetic correlation between blood metabolites and DVT and verified the causal relationship by MR analysis. Our study identified a set of suggestive candidate blood metabolites that showed an association with DVT. We hope that our findings will provide new insights into the pathogenesis and diagnosis of DVT in the future and serve as a basic resource for understanding the genetic mechanism of the effects of blood metabolites on DVT.

## Supplementary Information

Supplementary Tables.

## Data Availability

The datasets used and/or analyzed during the current study are available from the corresponding author on reasonable request. The GWAS dataset of DVT is available at GeneATLAS website, http://geneatlas.roslin.ed.ac.uk/. The GWAS dataset of human blood metabolites: http://metabolomics.helmholtz-muenchen.de/gwas/gwas_server/shin_et_al.metal.out.tar.gz.

## Abbreviations

DVT: Deep vein thrombosis
US: Ultrasound
LDSC: Linkage disequilibrium score regression
GWAS: Genome-wide association study
MR: Mendelian randomization
IVW: Inverse-variance weighted
FXIII: Factor XIII
5-HTP: 5-Hydroxytryptophan
SSRIs: Selective serotonin reuptake inhibitors
