# Supplementary material for: Large-scale genetic correlation scanning and causal association between deep vein thrombosis and human blood metabolites
Source: Sci Rep. 2022 May 12;12:7888. doi: 10.1038/s41598-022-12021-x (PMC9098636; doi:10.1038/s41598-022-12021-x)
Supplement: Supplementary file 1 — Supplementary Tables. [file 41598_2022_12021_MOESM1_ESM.docx]

**Large-scale genetic correlation scanning and causal association between deep** **vein thrombosis and human blood metabolites**

Pan Luo^1^, Jiawen Xu^2^, Shiqiang Cheng^3^, Ke Xu^1^,Wensen Jing^1^, Feng Zhang^3^, Peng Xu^*1^,

1 Department of Joint Surgery, HongHui Hospital, Xi’an Jiaotong University, Xi’an, Shanxi, 710054, China

2 Orthopedic Research Institute, Department of Orthopedics, West China Hospital, Sichuan University, 37# Guoxue Road, Chengdu, 610041, People’s Republic of China.

3 Key Laboratory of Trace Elements and Endemic Diseases, National Health and Family Planning Commission, School of Public Health, Health Science Center, Xi’an Jiao tong University, No.76 Yan Ta West Road, Xi’an 710061, People’s Republic of China

*Correspondence to:

Peng Xu, MD Department of Joint Surgery, HongHui Hospital, Xi’an Jiaotong University, Email: [sousou369@163.com](mailto:sousou369@163.com). Xi’an, Shanxi, 710054, China

**Supplementary Table 1: The genetic correlation results of the metabolites.**

| Blood metabolite ID |  | rg | se | z | p |
| --- | --- | --- | --- | --- | --- |
| M37112 | DVT | 0.2705 | 0.2809 | 0.9629 | 0.3356 |
| M37190 | DVT | -0.1803 | 0.2412 | -0.7477 | 0.4546 |
| M37202 | DVT | 0.2626 | 0.3284 | 0.7997 | 0.4239 |
| M37203 | DVT | 0.0873 | 0.1789 | 0.4878 | 0.6257 |
| M37459 | DVT | 0.8558 | 1.8563 | 0.461 | 0.6448 |
| M37506 | DVT | 0.1317 | 0.1156 | 1.1396 | 0.2545 |
| M38178 | DVT | -0.0146 | 0.152 | -0.0961 | 0.9234 |
| M38768 | DVT | -0.1873 | 0.1558 | -1.2024 | 0.2292 |
| M00053 | DVT | 0.0272 | 0.1348 | 0.2021 | 0.8399 |
| M00054 | DVT | -0.025 | 0.0808 | -0.3099 | 0.7566 |
| M00059 | DVT | -0.1758 | 0.1461 | -1.2032 | 0.2289 |
| M00060 | DVT | 0.0565 | 0.0647 | 0.8731 | 0.3826 |
| M00063 | DVT | 0.1047 | 0.2053 | 0.5099 | 0.6101 |
| M00064 | DVT | 0.0969 | 0.1645 | 0.589 | 0.5558 |
| M00542 | DVT | 0.3216 | 0.7599 | 0.4232 | 0.6722 |
| M00599 | DVT | -0.0372 | 0.1657 | -0.2247 | 0.8222 |
| M01105 | DVT | 0.0886 | 0.234 | 0.3787 | 0.7049 |
| M01123 | DVT | -0.224 | 0.2022 | -1.1079 | 0.2679 |
| M01284 | DVT | -0.0318 | 0.2558 | -0.1243 | 0.9011 |
| M01299 | DVT | 0.1046 | 0.1541 | 0.6788 | 0.4972 |
| M01303 | DVT | 0.3878 | 0.4858 | 0.7983 | 0.4247 |
| M01336 | DVT | 0.0852 | 0.101 | 0.8435 | 0.3989 |
| M01356 | DVT | -0.0987 | 0.1387 | -0.7116 | 0.4767 |
| M01358 | DVT | -0.0137 | 0.0943 | -0.1455 | 0.8843 |
| M01359 | DVT | 0.481 | 0.7442 | 0.6463 | 0.5181 |
| M01365 | DVT | -0.0151 | 0.15 | -0.1009 | 0.9197 |
| M01494 | DVT | -0.0388 | 0.1085 | -0.3574 | 0.7208 |
| M01508 | DVT | 0.019 | 0.1589 | 0.1193 | 0.905 |
| M01558 | DVT | 0.0691 | 0.0894 | 0.7727 | 0.4397 |
| M01564 | DVT | -0.0289 | 0.1013 | -0.2856 | 0.7752 |
| M01572 | DVT | -0.1497 | 0.2334 | -0.6415 | 0.5212 |
| M01573 | DVT | -0.1622 | 0.2136 | -0.7594 | 0.4476 |
| M01605 | DVT | -0.1145 | 0.261 | -0.4386 | 0.661 |
| M01638 | DVT | -0.8448 | 1.8701 | -0.4517 | 0.6515 |
| M01642 | DVT | -0.2137 | 0.1042 | -2.0508 | 0.0403 |
| M01644 | DVT | -0.1659 | 0.1069 | -1.5521 | 0.1206 |
| M01645 | DVT | -0.093 | 0.092 | -1.0104 | 0.3123 |
| M01649 | DVT | 0.244 | 0.1205 | 2.0242 | 0.043 |
| M01769 | DVT | -0.0165 | 0.1515 | -0.1092 | 0.913 |
| M01898 | DVT | 0.0613 | 0.1046 | 0.5862 | 0.5578 |
| M02132 | DVT | -0.0369 | 0.1137 | -0.3241 | 0.7459 |
| M02342 | DVT | -0.5666 | 0.3341 | -1.696 | 0.0899 |
| M02730 | DVT | 0.2232 | 0.3547 | 0.6292 | 0.5292 |
| M02734 | DVT | 0.1526 | 0.1026 | 1.4882 | 0.1367 |
| M03127 | DVT | 0.1156 | 0.1622 | 0.7131 | 0.4758 |
| M11438 | DVT | 0.0268 | 0.1889 | 0.1418 | 0.8872 |
| M12017 | DVT | -0.0467 | 0.1711 | -0.2728 | 0.785 |
| M12035 | DVT | -0.2154 | 0.1199 | -1.7963 | 0.0724 |
| M12129 | DVT | -0.019 | 0.1556 | -0.1218 | 0.903 |
| M12261 | DVT | 0.0531 | 0.2318 | 0.2289 | 0.8189 |
| M12593 | DVT | -0.2896 | 0.8667 | -0.3341 | 0.7383 |
| M12768 | DVT | -0.0337 | 0.1339 | -0.2516 | 0.8014 |
| M12774 | DVT | 0.0098 | 0.2214 | 0.0441 | 0.9648 |
| M15140 | DVT | 0.0918 | 0.1076 | 0.853 | 0.3937 |
| M15335 | DVT | 0.0113 | 0.3192 | 0.0355 | 0.9717 |
| M15488 | DVT | 0.1364 | 0.176 | 0.7753 | 0.4382 |
| M15500 | DVT | 0.1574 | 0.0645 | 2.4425 | 0.0146 |
| M15650 | DVT | 0.0904 | 0.1499 | 0.603 | 0.5465 |
| M15676 | DVT | 0.1678 | 0.1304 | 1.2866 | 0.1982 |
| M15677 | DVT | 0.1958 | 0.3878 | 0.505 | 0.6136 |
| M15990 | DVT | -0.3067 | 0.3419 | -0.8972 | 0.3696 |
| M15996 | DVT | 0.1041 | 0.2796 | 0.3724 | 0.7096 |
| M16816 | DVT | 0.241 | 0.2144 | 1.124 | 0.261 |
| M16821 | DVT | 0.1142 | 0.1626 | 0.7019 | 0.4827 |
| M16822 | DVT | 0.0473 | 0.171 | 0.2764 | 0.7822 |
| M16823 | DVT | -0.1446 | 0.2673 | -0.541 | 0.5885 |
| M17805 | DVT | 0.0034 | 0.2253 | 0.0149 | 0.9881 |
| M17807 | DVT | 0.5162 | 2.3168 | 0.2228 | 0.8237 |
| M17945 | DVT | -0.0222 | 0.103 | -0.2158 | 0.8292 |
| M18283 | DVT | 0.1993 | 0.2776 | 0.7179 | 0.4728 |
| M18349 | DVT | 0.0294 | 0.1195 | 0.2458 | 0.8058 |
| M18357 | DVT | -0.025 | 0.4045 | -0.0617 | 0.9508 |
| M18369 | DVT | 0.0839 | 0.1232 | 0.6811 | 0.4958 |
| M18476 | DVT | 0.3712 | 0.3717 | 0.9988 | 0.3179 |
| M18497 | DVT | 0.2405 | 0.2966 | 0.8109 | 0.4174 |
| M19364 | DVT | -0.0646 | 0.1761 | -0.3669 | 0.7137 |
| M19368 | DVT | -0.0568 | 0.2084 | -0.2723 | 0.7854 |
| M19934 | DVT | 0.1079 | 0.098 | 1.1013 | 0.2708 |
| M20489 | DVT | 0.244 | 0.1719 | 1.4194 | 0.1558 |
| M20675 | DVT | 0.1807 | 0.111 | 1.629 | 0.1033 |
| M21044 | DVT | 0.1672 | 0.2117 | 0.7901 | 0.4295 |
| M21049 | DVT | 0.3768 | 0.219 | 1.7208 | 0.0853 |
| M21127 | DVT | -0.0087 | 0.1138 | -0.0767 | 0.9388 |
| M21184 | DVT | 0.2552 | 0.6232 | 0.4094 | 0.6822 |
| M21188 | DVT | 0.0584 | 0.1596 | 0.366 | 0.7144 |
| M21630 | DVT | 0.0675 | 0.14 | 0.4823 | 0.6296 |
| M22030 | DVT | -0.0084 | 0.1357 | -0.0619 | 0.9507 |
| M22032 | DVT | 0.3687 | 0.4805 | 0.7674 | 0.4428 |
| M22130 | DVT | 0.0847 | 0.2808 | 0.3016 | 0.763 |
| M22138 | DVT | 0.1357 | 0.1922 | 0.7062 | 0.4801 |
| M22177 | DVT | -0.0887 | 0.0866 | -1.0251 | 0.3053 |
| M24074 | DVT | -0.0173 | 0.1336 | -0.1296 | 0.8969 |
| M25459 | DVT | -0.1635 | 0.1361 | -1.2013 | 0.2296 |
| M25599 | DVT | -0.1451 | 0.1655 | -0.8764 | 0.3808 |
| M27256 | DVT | 0.0863 | 0.14 | 0.6164 | 0.5376 |
| M27278 | DVT | 0.0475 | 0.2207 | 0.2151 | 0.8297 |
| M27672 | DVT | -0.0632 | 0.1969 | -0.3207 | 0.7485 |
| M27710 | DVT | 0.2639 | 0.2201 | 1.1986 | 0.2307 |
| M27718 | DVT | 0.3457 | 0.2518 | 1.3731 | 0.1697 |
| M27722 | DVT | -0.1944 | 0.3826 | -0.5082 | 0.6113 |
| M27738 | DVT | 0.1416 | 0.1835 | 0.7718 | 0.4402 |
| M31536 | DVT | 0.1855 | 0.2855 | 0.6499 | 0.5158 |
| M31591 | DVT | -0.1752 | 0.1745 | -1.0043 | 0.3152 |
| M31787 | DVT | -0.654 | 0.6164 | -1.0611 | 0.2886 |
| M32197 | DVT | 0.0494 | 0.1414 | 0.3492 | 0.7269 |
| M32315 | DVT | -0.2009 | 0.2811 | -0.7148 | 0.4747 |
| M32322 | DVT | 0.3043 | 0.1982 | 1.5353 | 0.1247 |
| M32338 | DVT | -0.0648 | 0.118 | -0.5491 | 0.5829 |
| M32339 | DVT | -0.0211 | 0.097 | -0.2175 | 0.8278 |
| M32348 | DVT | 0.0999 | 0.1025 | 0.9749 | 0.3296 |
| M32379 | DVT | -0.9273 | 4.8119 | -0.1927 | 0.8472 |
| M32388 | DVT | -0.6695 | 1.1386 | -0.588 | 0.5565 |
| M32393 | DVT | 0.3125 | 0.2755 | 1.1343 | 0.2566 |
| M32405 | DVT | -0.2199 | 0.2646 | -0.8312 | 0.4059 |
| M32412 | DVT | 0.1715 | 0.1766 | 0.9715 | 0.3313 |
| M32425 | DVT | 0.1367 | 0.2731 | 0.5004 | 0.6168 |
| M32452 | DVT | 0.0126 | 0.1521 | 0.0829 | 0.934 |
| M32489 | DVT | 0.0338 | 0.0911 | 0.3704 | 0.7111 |
| M32492 | DVT | -0.1305 | 0.0962 | -1.3559 | 0.1751 |
| M32497 | DVT | -0.0912 | 0.119 | -0.7669 | 0.4431 |
| M32504 | DVT | 0.0322 | 0.2771 | 0.1161 | 0.9076 |
| M32518 | DVT | -0.0599 | 0.1297 | -0.4615 | 0.6444 |
| M32549 | DVT | -0.3157 | 0.2 | -1.5781 | 0.1145 |
| M32560 | DVT | -0.1022 | 0.2546 | -0.4014 | 0.6882 |
| M32564 | DVT | -0.3843 | 0.3414 | -1.1257 | 0.2603 |
| M32578 | DVT | 0.3845 | 0.4036 | 0.9527 | 0.3408 |
| M32586 | DVT | -0.0439 | 0.2683 | -0.1635 | 0.8701 |
| M32587 | DVT | -0.3406 | 0.22 | -1.548 | 0.1216 |
| M32593 | DVT | 0.1317 | 0.215 | 0.6127 | 0.5401 |
| M32616 | DVT | 0.1045 | 0.2781 | 0.3756 | 0.7072 |
| M32632 | DVT | -0.1559 | 0.2357 | -0.6614 | 0.5083 |
| M32634 | DVT | -0.2181 | 0.105 | -2.0778 | 0.0377 |
| M32644 | DVT | -0.1558 | 0.0935 | -1.667 | 0.0955 |
| M32651 | DVT | -0.103 | 0.1647 | -0.625 | 0.5319 |
| M32672 | DVT | -0.2115 | 0.1757 | -1.2038 | 0.2287 |
| M32709 | DVT | 0.1605 | 0.1141 | 1.4061 | 0.1597 |
| M32729 | DVT | -0.1108 | 0.1034 | -1.0714 | 0.284 |
| M32735 | DVT | 0.2663 | 0.1914 | 1.391 | 0.1642 |
| M32740 | DVT | 0.0738 | 0.0892 | 0.8265 | 0.4085 |
| M32753 | DVT | -0.3302 | 0.4117 | -0.8019 | 0.4226 |
| M32754 | DVT | 0.0396 | 0.5733 | 0.0691 | 0.9449 |
| M32755 | DVT | -0.1166 | 0.219 | -0.5325 | 0.5944 |
| M32757 | DVT | -0.1 | 0.4731 | -0.2113 | 0.8326 |
| M32758 | DVT | -0.1953 | 0.8776 | -0.2225 | 0.8239 |
| M32761 | DVT | 0.0585 | 0.2566 | 0.2282 | 0.8195 |
| M32786 | DVT | -0.2562 | 0.2785 | -0.9202 | 0.3575 |
| M32787 | DVT | -0.2336 | 0.1986 | -1.1764 | 0.2394 |
| M32795 | DVT | 0.2898 | 0.5546 | 0.5226 | 0.6013 |
| M32800 | DVT | -0.0324 | 0.2419 | -0.1339 | 0.8935 |
| M32814 | DVT | -0.2904 | 0.1806 | -1.6076 | 0.1079 |
| M32838 | DVT | 0.3663 | 0.3151 | 1.1624 | 0.2451 |
| M32846 | DVT | 0.0489 | 0.3803 | 0.1285 | 0.8978 |
| M32854 | DVT | -0.4249 | 0.6137 | -0.6924 | 0.4887 |
| M32855 | DVT | -0.0409 | 0.1833 | -0.2232 | 0.8234 |
| M32857 | DVT | -0.2906 | 0.3724 | -0.7804 | 0.4352 |
| M32863 | DVT | 0.2236 | 0.1769 | 1.2642 | 0.2062 |
| M32867 | DVT | -0.2855 | 0.1414 | -2.019 | 0.0435 |
| M32910 | DVT | 0.1012 | 0.0983 | 1.0291 | 0.3034 |
| M33009 | DVT | 0.1537 | 0.364 | 0.4223 | 0.6728 |
| M33131 | DVT | 0.0171 | 0.1765 | 0.0969 | 0.9228 |
| M33140 | DVT | 0.3485 | 0.4889 | 0.7129 | 0.4759 |
| M33144 | DVT | 0.1126 | 0.2108 | 0.534 | 0.5933 |
| M33163 | DVT | 0.0106 | 0.3831 | 0.0276 | 0.978 |
| M33165 | DVT | 0.0894 | 0.1811 | 0.4936 | 0.6216 |
| M33192 | DVT | -0.5466 | 0.3514 | -1.5552 | 0.1199 |
| M33194 | DVT | -0.469 | 0.3567 | -1.3149 | 0.1885 |
| M33195 | DVT | -0.5734 | 0.8726 | -0.6571 | 0.5111 |
| M33204 | DVT | -0.2041 | 0.2192 | -0.9309 | 0.3519 |
| M33228 | DVT | -0.0397 | 0.1196 | -0.3316 | 0.7402 |
| M33250 | DVT | 0.0765 | 1.0136 | 0.0755 | 0.9399 |
| M33353 | DVT | 0.3855 | 0.2654 | 1.4527 | 0.1463 |
| M33380 | DVT | -0.0433 | 0.1872 | -0.2312 | 0.8172 |
| M33389 | DVT | -0.1661 | 0.1025 | -1.62 | 0.1052 |
| M33391 | DVT | 0.182 | 0.2043 | 0.891 | 0.3729 |
| M33415 | DVT | -0.1111 | 0.1034 | -1.0747 | 0.2825 |
| M33420 | DVT | -0.2676 | 0.4251 | -0.6295 | 0.529 |
| M33422 | DVT | 0.1402 | 0.1453 | 0.9652 | 0.3344 |
| M33441 | DVT | 0.0977 | 0.1405 | 0.6951 | 0.487 |
| M33447 | DVT | 0.236 | 0.2108 | 1.12 | 0.2627 |
| M33453 | DVT | 0.3138 | 0.2252 | 1.3934 | 0.1635 |
| M33488 | DVT | 0.0017 | 0.1815 | 0.0093 | 0.9926 |
| M33508 | DVT | 0.2124 | 0.1928 | 1.1014 | 0.2707 |
| M33509 | DVT | 0.2889 | 0.2117 | 1.3646 | 0.1724 |
| M33510 | DVT | 0.1647 | 0.1091 | 1.51 | 0.1311 |
| M33515 | DVT | 0.2376 | 0.1133 | 2.097 | 0.036 |
| M33609 | DVT | 0.156 | 0.19 | 0.8212 | 0.4115 |
| M33610 | DVT | -0.0221 | 0.1547 | -0.1427 | 0.8865 |
| M33633 | DVT | 0.0249 | 0.313 | 0.0795 | 0.9366 |
| M33637 | DVT | 0.1376 | 0.2508 | 0.5486 | 0.5833 |
| M33638 | DVT | 0.4658 | 0.357 | 1.3047 | 0.192 |
| M33652 | DVT | 0.7019 | 0.5527 | 1.2698 | 0.2041 |
| M33653 | DVT | 0.4522 | 0.3146 | 1.4376 | 0.1506 |
| M33666 | DVT | -0.3102 | 0.3009 | -1.0308 | 0.3027 |
| M33751 | DVT | -0.0009 | 0.2001 | -0.0045 | 0.9964 |
| M33782 | DVT | 0.1706 | 0.2268 | 0.7523 | 0.4519 |
| M33833 | DVT | 0.0443 | 0.3262 | 0.1359 | 0.8919 |
| M33835 | DVT | 0.1188 | 0.3166 | 0.3752 | 0.7075 |
| M33871 | DVT | -0.1774 | 0.219 | -0.81 | 0.418 |
| M33877 | DVT | 0.2373 | 0.2858 | 0.8302 | 0.4064 |
| M33883 | DVT | 0.3428 | 0.5737 | 0.5975 | 0.5502 |
| M33892 | DVT | 0.2035 | 0.1256 | 1.6201 | 0.1052 |
| M33901 | DVT | 0.193 | 0.3665 | 0.5266 | 0.5985 |
| M33910 | DVT | 0.3643 | 0.173 | 2.1053 | 0.0353 |
| M33936 | DVT | -0.021 | 0.1207 | -0.174 | 0.8619 |
| M33937 | DVT | 0.2996 | 0.3678 | 0.8145 | 0.4154 |
| M33941 | DVT | -0.0565 | 0.163 | -0.3466 | 0.7289 |
| M33955 | DVT | -0.1695 | 0.1467 | -1.1555 | 0.2479 |
| M33957 | DVT | -0.2017 | 0.1993 | -1.0119 | 0.3116 |
| M33960 | DVT | -0.0704 | 0.1614 | -0.4359 | 0.6629 |
| M33961 | DVT | -0.1362 | 0.3076 | -0.4428 | 0.6579 |
| M33969 | DVT | 0.0577 | 0.2175 | 0.2652 | 0.7909 |
| M33971 | DVT | 0.1324 | 0.329 | 0.4023 | 0.6875 |
| M33972 | DVT | 0.0398 | 0.2422 | 0.1642 | 0.8696 |
| M33973 | DVT | -0.2617 | 0.3263 | -0.802 | 0.4225 |
| M34040 | DVT | 0.0065 | 0.1354 | 0.0481 | 0.9617 |
| M34112 | DVT | -0.0757 | 0.1733 | -0.4368 | 0.6623 |
| M34214 | DVT | -0.1246 | 0.1422 | -0.8769 | 0.3806 |
| M34244 | DVT | -0.4936 | 0.1787 | -2.7624 | 0.0057 |
| M34283 | DVT | -0.1085 | 0.0991 | -1.0949 | 0.2735 |
| M34306 | DVT | -0.0187 | 0.9471 | -0.0197 | 0.9843 |
| M34314 | DVT | 0.1177 | 0.215 | 0.5473 | 0.5842 |
| M34327 | DVT | -0.1688 | 0.345 | -0.4892 | 0.6247 |
| M34329 | DVT | -0.3517 | 0.1827 | -1.9256 | 0.0541 |
| M34336 | DVT | 0.2084 | 0.2701 | 0.7715 | 0.4404 |
| M34338 | DVT | 0.0386 | 0.2126 | 0.1814 | 0.856 |
| M34339 | DVT | -0.3681 | 0.6018 | -0.6117 | 0.5408 |
| M34350 | DVT | -0.1142 | 0.2154 | -0.5304 | 0.5958 |
| M34359 | DVT | 0.1694 | 0.0972 | 1.7434 | 0.0813 |
| M34407 | DVT | -0.0078 | 0.1339 | -0.0586 | 0.9533 |
| M34416 | DVT | -0.385 | 0.1727 | -2.2299 | 0.0258 |
| M34420 | DVT | 0.3836 | 0.5158 | 0.7437 | 0.457 |
| M34441 | DVT | -0.1134 | 0.2399 | -0.4726 | 0.6365 |
| M34456 | DVT | -0.0931 | 0.1157 | -0.8041 | 0.4213 |
| M34469 | DVT | -0.2158 | 0.1866 | -1.1562 | 0.2476 |
| M34516 | DVT | -0.1579 | 0.1761 | -0.8966 | 0.3699 |
| M34533 | DVT | 0.7707 | 0.5034 | 1.5308 | 0.1258 |
| M34534 | DVT | 0.0448 | 0.2672 | 0.1675 | 0.8669 |
| M34535 | DVT | -0.2995 | 0.4108 | -0.7291 | 0.4659 |
| M34539 | DVT | 0.2274 | 0.1842 | 1.2344 | 0.2171 |
| M34674 | DVT | -0.2713 | 0.5547 | -0.4891 | 0.6248 |
| M34732 | DVT | 0.206 | 1.0476 | 0.1966 | 0.8441 |
| M34912 | DVT | -0.04 | 0.0962 | -0.4158 | 0.6775 |
| M35126 | DVT | -0.2384 | 0.2714 | -0.8783 | 0.3798 |
| M35127 | DVT | 0.0501 | 0.2212 | 0.2264 | 0.8209 |
| M35137 | DVT | 0.0007 | 0.099 | 0.0074 | 0.9941 |
| M35160 | DVT | 0.1153 | 0.1597 | 0.7218 | 0.4704 |
| M35186 | DVT | -0.0355 | 0.1579 | -0.2248 | 0.8222 |
| M35187 | DVT | -0.0668 | 0.2785 | -0.2399 | 0.8104 |
| M35189 | DVT | -0.0166 | 0.1546 | -0.1071 | 0.9147 |
| M35193 | DVT | 0.0664 | 0.1298 | 0.5116 | 0.6089 |
| M35253 | DVT | -0.1072 | 0.1431 | -0.7495 | 0.4536 |
| M35254 | DVT | -0.1046 | 0.1509 | -0.6932 | 0.4882 |
| M35257 | DVT | -0.1548 | 0.1663 | -0.9309 | 0.3519 |
| M35270 | DVT | -0.4281 | 0.2392 | -1.7896 | 0.0735 |
| M35305 | DVT | 0.1417 | 0.1973 | 0.7186 | 0.4724 |
| M35326 | DVT | -0.0854 | 0.1135 | -0.7526 | 0.4517 |
| M35331 | DVT | 0.0254 | 0.2037 | 0.1247 | 0.9008 |
| M35397 | DVT | -0.2052 | 0.1567 | -1.3094 | 0.1904 |
| M35439 | DVT | 0.014 | 0.1765 | 0.0792 | 0.9369 |
| M35464 | DVT | -0.0105 | 0.1468 | -0.0713 | 0.9431 |
| M35527 | DVT | 0.4355 | 0.3894 | 1.1183 | 0.2634 |
| M35551 | DVT | 0.6743 | 0.3078 | 2.1909 | 0.0285 |
| M35628 | DVT | 0.0078 | 0.2777 | 0.0282 | 0.9775 |
| M35631 | DVT | -0.2317 | 0.1547 | -1.498 | 0.1341 |
| M35669 | DVT | -0.0562 | 0.1503 | -0.374 | 0.7084 |
| M35675 | DVT | -0.0939 | 0.0988 | -0.9499 | 0.3422 |
| M35678 | DVT | 0.0412 | 0.2545 | 0.1618 | 0.8714 |
| M35754 | DVT | -0.0333 | 0.1014 | -0.3283 | 0.7427 |
| M35977 | DVT | -0.1408 | 0.6732 | -0.2092 | 0.8343 |
| M35978 | DVT | 0.1885 | 0.1706 | 1.1046 | 0.2693 |
| M36103 | DVT | -0.1889 | 0.2044 | -0.9242 | 0.3554 |
| M36131 | DVT | -0.2576 | 0.3582 | -0.7192 | 0.472 |
| M36399 | DVT | 0.8371 | 0.8965 | 0.9337 | 0.3505 |
| M36515 | DVT | 0.0597 | 0.1316 | 0.454 | 0.6498 |
| M36553 | DVT | -0.1043 | 0.109 | -0.9566 | 0.3387 |
| M36585 | DVT | 0.2082 | 0.4357 | 0.4778 | 0.6328 |
| M36673 | DVT | -0.2179 | 0.5332 | -0.4087 | 0.6827 |
| M36754 | DVT | -0.015 | 0.2099 | -0.0715 | 0.943 |
| M36756 | DVT | -0.1914 | 0.2287 | -0.8369 | 0.4026 |
| M36802 | DVT | 0.0889 | 0.1421 | 0.6253 | 0.5318 |
| M36808 | DVT | -0.2561 | 0.2578 | -0.9934 | 0.3205 |
| M36850 | DVT | 0.1154 | 0.2567 | 0.4497 | 0.653 |
| M37058 | DVT | 0.0662 | 0.0849 | 0.7797 | 0.4356 |
| M37097 | DVT | 0.009 | 0.1715 | 0.0524 | 0.9582 |

**Supplementary Table 2：Metabolite ID interpretation**

| metabolonID | metabolonDescription |
| --- | --- |
| M00053 | glutamine |
| M00054 | tryptophan |
| M00059 | histidine |
| M00060 | leucine |
| M00063 | cholesterol |
| M00064 | phenylalanine |
| M00513 | creatinine |
| M00527 | lactate |
| M00541 | 4-hydroxyphenylacetate |
| M00542 | 3-hydroxybutyrate (BHBA) |
| M00553 | cotinine |
| M00555 | adenosine |
| M00569 | caffeine |
| M00575 | arabinose |
| M00577 | fructose |
| M00584 | mannose |
| M00599 | pyruvate |
| M00606 | uridine |
| M01105 | linoleate (18:2n6) |
| M01107 | allantoin |
| M01110 | arachidonate (20:4n6) |
| M01114 | deoxycholate |
| M01121 | margarate (17:0) |
| M01123 | inosine |
| M01125 | isoleucine |
| M01284 | threonine |
| M01299 | tyrosine |
| M01301 | lysine |
| M01302 | methionine |
| M01303 | malate |
| M01336 | palmitate (16:0) |
| M01356 | nonadecanoate (19:0) |
| M01358 | stearate (18:0) |
| M01359 | oleate (18:1n9) |
| M01361 | pentadecanoate (15:0) |
| M01365 | myristate (14:0) |
| M01444 | pipecolate |
| M01481 | inositol 1-phosphate (I1P) |
| M01493 | ornithine |
| M01494 | 5-oxoproline |
| M01508 | pantothenate |
| M01515 | salicylate |
| M01558 | 4-acetamidobutanoate |
| M01561 | alpha-tocopherol |
| M01564 | citrate |
| M01572 | glycerate |
| M01573 | guanosine |
| M01585 | N-acetylalanine |
| M01604 | urate |
| M01605 | ursodeoxycholate |
| M01638 | arginine |
| M01640 | ascorbate (Vitamin C) |
| M01644 | heptanoate (7:0) |
| M01645 | laurate (12:0) |
| M01649 | valine |
| M01670 | urea |
| M01712 | cortisol |
| M01769 | cortisone |
| M01827 | riboflavin (Vitamin B2) |
| M01898 | proline |
| M02132 | citrulline |
| M02137 | biliverdin |
| M02342 | serotonin (5HT) |
| M02730 | gamma-glutamylglutamine |
| M02734 | gamma-glutamyltyrosine |
| M03127 | hypoxanthine |
| M03141 | betaine |
| M03147 | xanthine |
| M10642 | caprate (10:0) |
| M11438 | phosphate |
| M12017 | 3-methoxytyrosine |
| M12032 | 4-acetamidophenol |
| M12035 | pelargonate (9:0) |
| M12067 | undecanoate (11:0) |
| M12122 | naproxen |
| M12129 | beta-hydroxyisovalerate |
| M12261 | taurodeoxycholate |
| M12593 | X-02973 |
| M12626 | X-03003 |
| M12768 | X-03088 |
| M12770 | X-03090 |
| M12774 | X-03094 |
| M15122 | glycerol |
| M15140 | kynurenine |
| M15335 | mannitol |
| M15365 | glycerol 3-phosphate (G3P) |
| M15488 | acetylphosphate |
| M15500 | carnitine |
| M15506 | choline |
| M15630 | N-acetylornithine |
| M15650 | N1-methyladenosine |
| M15676 | 3-methyl-2-oxovalerate |
| M15677 | 3-methylhistidine |
| M15749 | 3-phenylpropionate (hydrocinnamate) |
| M15753 | hippurate |
| M15778 | benzoate |
| M15958 | phenylacetate |
| M15964 | arabitol |
| M15990 | glycerophosphorylcholine (GPC) |
| M15996 | aspartate |
| M16634 | X-04357 |
| M16816 | X-04494 |
| M16818 | X-04495 |
| M16821 | X-04498 |
| M16822 | X-04499 |
| M16823 | X-04500 |
| M17059 | X-04621 |
| M17799 | ibuprofen |
| M17805 | dihomo-linoleate (20:2n6) |
| M17807 | X-18601 |
| M17945 | 2-hydroxystearate |
| M18037 | metoprolol |
| M18254 | paraxanthine |
| M18281 | 2-hydroxyhippurate (salicylurate) |
| M18283 | X-05426 |
| M18335 | quinate |
| M18349 | indolelactate |
| M18357 | glycylvaline |
| M18369 | gamma-glutamylleucine |
| M18392 | theobromine |
| M18394 | theophylline |
| M18467 | eicosapentaenoate (EPA; 20:5n3) |
| M18474 | estrone 3-sulfate |
| M18476 | glycocholate |
| M18477 | glycodeoxycholate |
| M18494 | taurochenodeoxycholate |
| M18497 | taurocholate |
| M18929 | X-05907 |
| M19323 | docosahexaenoate (DHA; 22:6n3) |
| M19324 | 1-stearoylglycerophosphoinositol |
| M19362 | X-06226 |
| M19363 | X-06227 |
| M19364 | X-06246 |
| M19368 | X-06267 |
| M19396 | X-06307 |
| M19414 | X-06350 |
| M19415 | X-06351 |
| M19934 | myo-inositol |
| M20489 | glucose |
| M20675 | 1,5-anhydroglucitol (1,5-AG) |
| M20699 | erythritol |
| M21044 | 2-hydroxybutyrate (AHB) |
| M21047 | 3-methyl-2-oxobutyrate |
| M21049 | 1,6-anhydroglucose |
| M21127 | 1-palmitoylglycerol (1-monopalmitin) |
| M21151 | saccharin |
| M21184 | 1-oleoylglycerol (1-monoolein) |
| M21188 | 1-stearoylglycerol (1-monostearin) |
| M21630 | X-08402 |
| M22030 | 2-hydroxyisobutyrate |
| M22032 | X-08766 |
| M22116 | 4-methyl-2-oxopentanoate |
| M22130 | phenyllactate (PLA) |
| M22138 | homocitrulline |
| M22175 | aspartylphenylalanine |
| M22177 | levulinate (4-oxovalerate) |
| M22189 | palmitoylcarnitine |
| M22481 | X-08988 |
| M22548 | X-09026 |
| M22649 | X-09108 |
| M22842 | cholate |
| M24074 | X-09706 |
| M25459 | X-10395 |
| M25599 | X-10429 |
| M27256 | X-10500 |
| M27273 | X-10506 |
| M27278 | X-10510 |
| M27447 | 1-linoleoylglycerol (1-monolinolein) |
| M27513 | indoleacetate |
| M27531 | hyodeoxycholate |
| M27672 | 3-indoxyl sulfate |
| M27710 | N-acetylglycine |
| M27716 | bilirubin (Z,Z) |
| M27718 | creatine |
| M27722 | erythrose |
| M27728 | glycerol 2-phosphate |
| M27738 | threonate |
| M28354 | X-10675 |
| M30805 | X-10810 |
| M31453 | cysteine |
| M31454 | cystine |
| M31522 | pyroglutamylglycine |
| M31536 | N-(2-furoyl)glycine |
| M31548 | DSGEGDFXAEGGGVR* |
| M31555 | pyridoxate |
| M31591 | androsterone sulfate |
| M31787 | 3-carboxy-4-methyl-5-propyl-2-furanpropanoate (CMPF) |
| M32197 | 3-(4-hydroxyphenyl)lactate |
| M32198 | acetylcarnitine |
| M32315 | serine |
| M32319 | trans-4-hydroxyproline |
| M32322 | glutamate |
| M32328 | hexanoylcarnitine |
| M32338 | glycine |
| M32339 | alanine |
| M32346 | glycochenodeoxycholate |
| M32348 | 2-aminobutyrate |
| M32379 | scyllo-inositol |
| M32388 | dodecanedioate |
| M32393 | gamma-glutamylvaline |
| M32397 | 3-hydroxy-2-ethylpropionate |
| M32398 | sebacate (decanedioate) |
| M32401 | trigonelline (N'-methylnicotinate) |
| M32405 | indolepropionate |
| M32412 | butyrylcarnitine |
| M32418 | myristoleate (14:1n5) |
| M32425 | dehydroisoandrosterone sulfate (DHEA-S) |
| M32445 | 3-methylxanthine |
| M32452 | propionylcarnitine |
| M32455 | linoleamide (18:2n6) |
| M32458 | oleamide |
| M32489 | caproate (6:0) |
| M32492 | caprylate (8:0) |
| M32497 | 10-undecenoate (11:1n1) |
| M32504 | docosapentaenoate (n3 DPA; 22:5n3) |
| M32518 | X-11204 |
| M32549 | X-02269 |
| M32553 | phenol sulfate |
| M32557 | X-06126 |
| M32560 | X-07765 |
| M32564 | X-11247 |
| M32578 | X-11261 |
| M32586 | bilirubin (E,E)* |
| M32587 | X-02249 |
| M32593 | heme* |
| M32616 | X-11299 |
| M32632 | X-11315 |
| M32634 | X-11317 |
| M32635 | 1-linoleoylglycerophosphoethanolamine* |
| M32644 | X-11327 |
| M32651 | X-11334 |
| M32654 | 3-dehydrocarnitine* |
| M32672 | pyroglutamine* |
| M32675 | C-glycosyltryptophan* |
| M32689 | X-11372 |
| M32691 | X-11374 |
| M32698 | X-11381 |
| M32709 | X-03056 |
| M32729 | X-11412 |
| M32735 | X-01911 |
| M32739 | X-11422 |
| M32740 | X-11423 |
| M32753 | X-09789 |
| M32754 | X-11437 |
| M32755 | X-11438 |
| M32757 | X-11440 |
| M32758 | X-11441 |
| M32759 | X-11442 |
| M32761 | X-11444 |
| M32762 | X-11445 |
| M32769 | X-11452 |
| M32786 | X-11469 |
| M32787 | X-11470 |
| M32795 | X-11478 |
| M32800 | X-11483 |
| M32802 | X-11485 |
| M32808 | X-11491 |
| M32814 | X-11497 |
| M32836 | HWESASXX* |
| M32838 | X-11521 |
| M32846 | X-11529 |
| M32847 | X-11530 |
| M32854 | X-11537 |
| M32855 | X-11538 |
| M32857 | X-11540 |
| M32863 | X-11546 |
| M32867 | X-11550 |
| M32869 | X-11552 |
| M32910 | X-11593 |
| M32980 | adrenate (22:4n6) |
| M33009 | homostachydrine* |
| M33084 | ADSGEGDFXAEGGGVR* |
| M33085 | hydroxypioglitazone* |
| M33131 | X-11786 |
| M33132 | X-11787 |
| M33137 | X-11792 |
| M33138 | X-11793 |
| M33139 | pioglitazone* |
| M33140 | X-11795 |
| M33144 | X-11799 |
| M33150 | X-11805 |
| M33154 | X-11809 |
| M33163 | X-11818 |
| M33165 | X-11820 |
| M33173 | 2-hydroxyacetaminophen sulfate* |
| M33178 | 2-methoxyacetaminophen sulfate* |
| M33183 | X-11838 |
| M33188 | X-11843 |
| M33190 | X-11845 |
| M33192 | X-11847 |
| M33194 | X-11849 |
| M33195 | X-11850 |
| M33197 | X-11852 |
| M33203 | X-11858 |
| M33204 | X-11859 |
| M33221 | X-11876 |
| M33225 | X-11880 |
| M33228 | 1-arachidonoylglycerophosphocholine* |
| M33230 | 1-palmitoleoylglycerophosphocholine* |
| M33250 | X-11905 |
| M33353 | X-12007 |
| M33359 | X-12013 |
| M33363 | gamma-glutamylmethionine* |
| M33364 | gamma-glutamylthreonine* |
| M33380 | X-12029 |
| M33384 | salicyluric glucuronide* |
| M33389 | X-12038 |
| M33390 | X-12039 |
| M33391 | X-12040 |
| M33408 | X-12056 |
| M33415 | X-12063 |
| M33420 | gamma-tocopherol |
| M33422 | gamma-glutamylphenylalanine |
| M33423 | p-acetamidophenylglucuronide |
| M33441 | isobutyrylcarnitine |
| M33442 | pseudouridine |
| M33443 | valerate |
| M33447 | palmitoleate (16:1n7) |
| M33453 | alpha-ketoglutarate |
| M33477 | erythronate* |
| M33488 | lathosterol |
| M33507 | X-12092 |
| M33508 | X-12093 |
| M33509 | X-12094 |
| M33510 | X-12095 |
| M33515 | X-12100 |
| M33531 | X-12116 |
| M33587 | eicosenoate (20:1n9 or 11) |
| M33609 | X-12188 |
| M33610 | X-12189 |
| M33616 | X-12195 |
| M33627 | X-12206 |
| M33633 | X-12212 |
| M33637 | X-12216 |
| M33638 | X-12217 |
| M33652 | X-12230 |
| M33653 | X-12231 |
| M33658 | X-12236 |
| M33666 | X-12244 |
| M33675 | X-12253 |
| M33683 | X-12261 |
| M33685 | X-12263 |
| M33751 | X-12329 |
| M33782 | X-10346 |
| M33801 | ADpSGEGDFXAEGGGVR* |
| M33821 | 1-eicosatrienoylglycerophosphocholine* |
| M33822 | 1-docosahexaenoylglycerophosphocholine* |
| M33833 | X-12405 |
| M33835 | X-12407 |
| M33864 | X-12428 |
| M33871 | 1-eicosadienoylglycerophosphocholine* |
| M33877 | X-12435 |
| M33883 | X-12441 |
| M33884 | X-12442 |
| M33885 | X-12443 |
| M33892 | X-12450 |
| M33901 | X-12456 |
| M33910 | X-12465 |
| M33935 | piperine |
| M33936 | octanoylcarnitine |
| M33937 | alpha-hydroxyisovalerate |
| M33939 | N-acetylthreonine |
| M33941 | decanoylcarnitine |
| M33955 | 1-palmitoylglycerophosphocholine |
| M33957 | 1-heptadecanoylglycerophosphocholine |
| M33960 | 1-oleoylglycerophosphocholine |
| M33961 | 1-stearoylglycerophosphocholine |
| M33968 | 5-dodecenoate (12:1n7) |
| M33969 | stearidonate (18:4n3) |
| M33971 | 10-heptadecenoate (17:1n7) |
| M33972 | 10-nonadecenoate (19:1n9) |
| M33973 | epiandrosterone sulfate |
| M34035 | linolenate [alpha or gamma; (18:3n3 or 6)] |
| M34040 | X-12510 |
| M34062 | X-12524 |
| M34102 | X-12539 |
| M34106 | bilirubin (E,Z or Z,E)* |
| M34109 | metoprolol acid metabolite* |
| M34112 | X-12544 |
| M34123 | X-12556 |
| M34214 | 1-arachidonoylglycerophosphoinositol* |
| M34221 | X-12627 |
| M34244 | X-12644 |
| M34245 | X-12645 |
| M34283 | asparagine |
| M34289 | X-12680 |
| M34306 | X-12696 |
| M34314 | X-12704 |
| M34321 | X-12711 |
| M34322 | X-12712 |
| M34327 | X-12717 |
| M34329 | X-12719 |
| M34336 | X-12726 |
| M34338 | X-12728 |
| M34339 | X-12729 |
| M34344 | X-12734 |
| M34350 | X-12740 |
| M34359 | X-12749 |
| M34365 | 3-(cystein-S-yl)acetaminophen* |
| M34369 | X-12759 |
| M34384 | stachydrine |
| M34389 | 1-methylxanthine |
| M34390 | 7-methylxanthine |
| M34395 | 1-methylurate |
| M34400 | 1,7-dimethylurate |
| M34404 | 1,3,7-trimethylurate |
| M34407 | isovalerylcarnitine |
| M34409 | stearoylcarnitine |
| M34416 | 1-stearoylglycerophosphoethanolamine |
| M34419 | 1-linoleoylglycerophosphocholine |
| M34420 | bradykinin, des-arg(9) |
| M34441 | X-12771 |
| M34453 | X-12776 |
| M34456 | gamma-glutamylisoleucine* |
| M34469 | X-12786 |
| M34481 | X-12798 |
| M34499 | X-12816 |
| M34513 | X-12830 |
| M34516 | X-12833 |
| M34527 | X-12844 |
| M34530 | X-12847 |
| M34533 | X-12850 |
| M34534 | laurylcarnitine |
| M34535 | X-12851 |
| M34539 | X-12855 |
| M34674 | X-12990 |
| M34732 | isovalerate |
| M34761 | X-13069 |
| M34826 | X-13134 |
| M34878 | X-13183 |
| M34912 | X-13215 |
| M35072 | X-13372 |
| M35114 | 7-methylguanine |
| M35126 | phenylacetylglutamine |
| M35127 | pro-hydroxy-pro |
| M35137 | N2,N2-dimethylguanosine |
| M35159 | cysteine-glutathione disulfide |
| M35160 | oleoylcarnitine |
| M35186 | 1-arachidonoylglycerophosphoethanolamine* |
| M35187 | X-13429 |
| M35189 | X-13431 |
| M35193 | X-13435 |
| M35240 | X-13477 |
| M35253 | 2-palmitoylglycerophosphocholine* |
| M35254 | 2-oleoylglycerophosphocholine* |
| M35255 | 2-stearoylglycerophosphocholine* |
| M35257 | 2-linoleoylglycerophosphocholine* |
| M35270 | X-13496 |
| M35305 | 1-palmitoylglycerophosphoinositol* |
| M35320 | catechol sulfate |
| M35322 | hydroquinone sulfate |
| M35326 | X-13548 |
| M35327 | X-13549 |
| M35331 | X-13553 |
| M35397 | X-13619 |
| M35422 | X-13640 |
| M35428 | tiglyl carnitine |
| M35431 | 2-methylbutyroylcarnitine |
| M35433 | hydroxyisovaleroyl carnitine |
| M35439 | glutaroyl carnitine |
| M35451 | X-13658 |
| M35464 | X-13671 |
| M35472 | 2-tetradecenoyl carnitine |
| M35508 | X-13699 |
| M35527 | 4-hydroxyhippurate |
| M35551 | X-13741 |
| M35626 | 1-myristoylglycerophosphocholine |
| M35628 | 1-oleoylglycerophosphoethanolamine |
| M35631 | 1-palmitoylglycerophosphoethanolamine |
| M35635 | 3-(3-hydroxyphenyl)propionate |
| M35669 | tetradecanedioate |
| M35675 | 2-hydroxypalmitate |
| M35678 | hexadecanedioate |
| M35718 | dihomo-linolenate (20:3n3 or n6) |
| M35754 | X-13859 |
| M35854 | threitol |
| M35977 | X-14056 |
| M35978 | X-14057 |
| M36009 | X-14086 |
| M36095 | thymol sulfate |
| M36097 | 4-acetaminophen sulfate |
| M36098 | 4-vinylphenol sulfate |
| M36099 | 4-ethylphenylsulfate |
| M36103 | p-cresol sulfate |
| M36115 | X-14189 |
| M36131 | X-14205 |
| M36134 | X-14208 |
| M36230 | X-14304 |
| M36300 | X-14374 |
| M36376 | X-14450 |
| M36394 | X-14468 |
| M36399 | X-14473 |
| M36468 | X-14541 |
| M36515 | X-14588 |
| M36552 | X-14625 |
| M36553 | X-14626 |
| M36559 | X-14632 |
| M36585 | X-14658 |
| M36589 | X-14662 |
| M36590 | X-14663 |
| M36593 | 2-linoleoylglycerophosphoethanolamine* |
| M36673 | X-14745 |
| M36738 | gamma-glutamylglutamate |
| M36754 | octadecanedioate |
| M36756 | leucylleucine |
| M36776 | 7-alpha-hydroxy-3-oxo-4-cholestenoate (7-Hoca) |
| M36802 | n-Butyl Oleate |
| M36808 | dimethylarginine (SDMA + ADMA) |
| M36848 | 3-ethylphenylsulfate* |
| M36850 | taurolithocholate 3-sulfate |
| M37004 | X-14977 |
| M37033 | carbamazepine* |
| M37058 | succinylcarnitine |
| M37097 | tryptophan betaine |
| M37104 | cyclo(leu-pro) |
| M37112 | chiro-inositol |
| M37190 | 5alpha-androstan-3beta,17beta-diol disulfate |
| M37198 | 5alpha-pregnan-3beta,20alpha-diol disulfate |
| M37202 | 4-androsten-3beta,17beta-diol disulfate 1* |
| M37203 | 4-androsten-3beta,17beta-diol disulfate 2* |
| M37253 | 2-hydroxyglutarate |
| M37459 | ergothioneine |
| M37506 | palmitoyl sphingomyelin |
| M38150 | phenylalanylphenylalanine |
| M38178 | cis-4-decenoyl carnitine |
| M38658 | atenolol |
| M38768 | 15-methylpalmitate (isobar with 2-methylpalmitate) |
| M39270 | 1-palmitoylplasmenylethanolamine* |
| M39379 | glycoursodeoxycholate |
